# Enhancement of ε-poly-l-lysine (ε-PL) production by a novel producer *Bacillus cereus* using metabolic precursors and glucose feeding

**DOI:** 10.1007/s13205-015-0291-8

**Published:** 2015-03-03

**Authors:** Anuj H. Chheda, Madhavi R. Vernekar

**Affiliations:** School of Biotechnology and Bioinformatics, D. Y. Patil University, C.B.D Belapur, Navi Mumbai, 400614 Maharashtra India

**Keywords:** ε-Poly-l-lysine, Fermentation, Citric acid, l-Aspartic acid, Metabolic precursors, TCA cycle

## Abstract

Epsilon poly-l-lysine (ε-PL) is a homo-biopolymer with approximately 25–30 l-lysine residues. It is a promising natural biopolymer widely used in food and pharmaceutical industry. The present work reports enhanced production of ε-PL with a novel producer *Bacillus cereus* using amino acids and TCA cycle intermediates in the fermentation medium. Among the various amino acids and TCA cycle intermediates tested 2 mM l-aspartic acid and 5 mM citric acid gave ε-PL yield of 145.5 and 230 mg/L, respectively. A combination of citric acid after 24 h and l-aspartic acid after 36 h improved ε-PL yield from 85 mg/L (control) to 335 mg/L. Glucose feeding strategy along with metabolic precursors was employed which further enhanced ε-PL yield to 565 mg/L. Thus, more than sixfold increase in ε-PL yield was achieved suggesting the potential of *Bacillus cereus* as a novel ε-PL producer.

## Introduction

ε-Poly-l-lysine (ε-PL) is a basic homo-poly-amino acid characterized by a peptide bond between ε-amino and α-carboxyl groups of l-lysine. It is a secondary metabolite, mainly produced by bacteria belonging to the family of *Streptomycetaceae* (Shima and Sakai 1977, 1981a, b). ε-PL possess antimicrobial activity against most Gram positive and Gram negative bacteria, fungi, yeast, etc. (Shima et al. 1984). It is water soluble, biodegradable, edible and nontoxic (Shih et al. 2004, 2006). Currently ε-PL is used as a natural food preservative in Japan, South Korea, USA and other countries. Apart from its use in food industry, it finds numerous applications in pharmaceutical industry as a drug carrier, as nanoparticles, as gene carrier, as liposomes, as interferon inducer, as lipase inhibitor, as hydrogel, as coating material, etc. (Bankar and Singhal 2013). Considering its wide array of applications, ε-PL has become a molecule of great interest and is gaining a lot of importance.

Tricarboxylic acid (TCA) cycle is a critical catabolic pathway which provides important precursors for the biosynthesis of amino acids, nucleic acids, lipids and polysaccharides. ε-PL is a linear polymer synthesized from l-lysine monomers by the formation of amide bonds between ε-amino and α-carboxyl groups. For biosynthesis of ε-PL from *Streptomyces albulus*, external lysine is used as a direct precursor (Shima et al. 1983). However, strains that produce relatively low molecular weight ε-PLs do not utilize external lysine and polymer production is reduced on addition of large amount of external lysine (Saimura et al. 2008). In bacteria lysine is synthesized through the diaminopimelate pathway (DAP). Diaminopimelic acid is formed via aspartate produced by transamination of oxaloacetate. Studies suggest that addition of citric acid to the production medium facilitates the conversion of oxaloacetate to aspartate by inhibiting the cycle forming reaction to citrate (Bankar and Singhal 2011). ε-PL production has been achieved from glucose in batch and fedbatch systems by *Streptomyces albulus* S 410 (Kahar et al. 2001), *Streptomyces albulus* IFO 14147 (Shih et al. 2006) and *Kitasatospora* sp. MY 5–36 (Zhang et al. 2010). Industrially, ε-PL is produced by aerobic fermentation with *Streptomyces albulus* (Shima and Sakai 1977, 1981a, b; Shima et al. 1984). But the major problem of using *Streptomyces albulus* is enzymatic and pH-induced degradation of secreted ε-PL in the culture medium (Hirohara et al. 2006). Attempts have been made in using alternative organisms such as *Streptomyces diastatochromogenes* CGMCC3145 (Wang et al. 2011), *Streptomyces aureofaciens* (Takehara et al. 2010), *Streptomyces noursei* NRRL 5126 (Banker and Singhal 2010), *Streptomyces griseofuscus* (Li et al. 2010), *Kitasatospora* sp. MY 5–36 (Zhang et al. 2010), *Kitasatospora kifunense* (Kobayashi and Nishikawa 2007), *Kitasatospora* sp. PL6 (Ouyang et al. 2006). However, there are very few research papers exploring bacteria as ε-PL producer (El-sersy et al. 2012; Shukla and Mishra 2013).

In earlier work we have reported a promising ε-PL producer, identified as *Bacillus cereus* (Chedda and Vernekar 2014). The present work involves improving the yield of ε-PL from *Bacillus cereus* using various metabolic precursors such as amino acids and TCA cycle intermediates. In addition, an attempt has been made to study fed batch fermentation for ε-PL production by addition of glucose intermittently in the fermentation medium with metabolic precursors.

## Materials and methods

### Chemicals

Unless otherwise stated, chemicals were purchased from M/S Hi-Media Limited Mumbai, and were of highest purity available. ε-PL was procured from Zhengzhou Bainafo Bioengineering Co. Ltd., China.

### Growth and maintenance of *Bacillus cereus*

ε-Poly-l-lysine producing strain *Bacillus cereus* was isolated from the hill region in CBD-Belapur, Navi Mumbai, India (Chedda and Vernekar 2014). It was maintained on a medium containing sodium caseinate 2 g/L, l-asparagine 0.1 g/L, sodium propionate 4 g/L, K_2_HPO_4_ 0.5 g/L, MgSO_4_.7H_2_O 0.1 g/L, FeSO_4_.7H_2_O 0.001 g/L and agar 15 g/L. 5 mL of glycerol was added to the above medium. The slants were incubated at 37 °C for 4 days and then stored at 4 °C.

Experiments were carried out in 100-mL Erlenmeyer flasks with 25 mL of production medium with following components (g/L): yeast extract, 10; glucose, 50; (NH_4_)_2_SO_4_, 15; MgSO_4_, 0.5; K_2_HPO_4_, 0.8; KH_2_PO_4_; 1.4, FeSO_4_, 0.04; ZnSO_4_, 0.04. The pH of the medium was adjusted to 6.8 with 1 N NaOH before sterilization (Chedda and Vernekar 2014). 4 % (v/v) of a 48-h-old culture (approximately 8.9 × 10^8^ cells/mL) was used as inoculum. Shake flask cultures of the organism were incubated at temperature 32 ± 2 °C with continuous agitation at 150 rpm for 96 h. These fermentation parameters were kept uniform for all the studies conducted. All experiments were carried out in triplicates.

### Analysis of ε-PL

Samples were withdrawn aseptically for analysis at regular intervals. The broth was centrifuged (10,000*g*, 10 min) and ε-PL concentration was measured in the supernatant using the method of Shen et al. (1984), which is based on selective binding of trypan blue with ε-PL. 1 mL of supernatant along with 2.88 mL of 0.1 mM phosphate buffer (pH 7) and 120 µL of trypan blue solution (1 mg/mL) were mixed thoroughly and incubated at 37 °C for 60 min. The mixture was centrifuged (10,000*g*, 10 min). The supernatant obtained was collected and absorbance was measured at 580 nm on UV–VIS spectrophotometer. A standard curve was derived from measurements with known amounts (0–50 mg/L) of ε-PL.

### Effect of amino acids on ε-PL production

Effect of Asp family amino acids on ε-PL production was studied. The amino acids tested were l-aspartic acid, l-methionine, l-lysine and l-threonine. Each amino acid was added at a concentration of 2 mM in 100-mL Erlenmeyer flasks with 25 mL of production medium. No amino acid was added in the control flask. Further to optimize the concentrations of selected amino acids viz, l-aspartic acid and l-lysine on ε-PL production, their concentrations were varied from 0.5 to 4 mM.

### Effect of precursors on ε-PL production

To study the effect of precursors on ε-PL production, various TCA cycle intermediates such as citric acid, α-ketoglutaric acid, oxaloacetic acid, fumaric acid, malic acid and succinic acid were tested at 10 mM concentration. Further screening was done with citric acid, α-ketoglutaric acid and oxaloacetic acid at a concentration between 2 and 20 mM.

### Optimization of time of addition of l-aspartic acid and citric acid on ε-PL production

The time of addition of l-aspartic acid and citric acid was optimized by supplementing the production medium with 2 mM l-aspartic acid and 5 mM citric acid, respectively, at different time intervals 12, 24, 36, 48 and 60 h from the start of fermentation process.

### Effect of intermittent feeding of glucose on ε-PL production

Effect of intermittent feeding of glucose on ε-PL production was studied by carrying out fedbatch fermentation at shake flask level. Experiments were carried out in 100-mL Erlenmeyer flasks with 25 mL of production medium inoculated with 4 % (v/v) of a 48-h-old culture. Shake flask cultures of the organism were incubated at temperature 32 ± 2 °C with continuous agitation at 150 rpm for 96 h. Glucose feeding was started after 24 h of fermentation. In the first set, 1 mL of glucose (10 g/L) was incorporated every 12 h in the production medium. No intermittent glucose addition was done in the control flask. In the second set synergistic effect of glucose-fedbatch fermentation along with metabolic precursors was studied. In this set, 1 mL (10 g/L) glucose was incorporated every 12 h in the production medium in which 5 mM citric acid was added after 24 h and 2 mM l-aspartic acid was added after 36 h, respectively. No glucose or metabolic precursors were added in the control flask. Samples from each flask were withdrawn after every 12 h and analyzed for ε-PL production and growth of *Bacillus cereus*.

## Results and discussion

### Effect of amino acids on ε-PL production

**Fig. 1:**
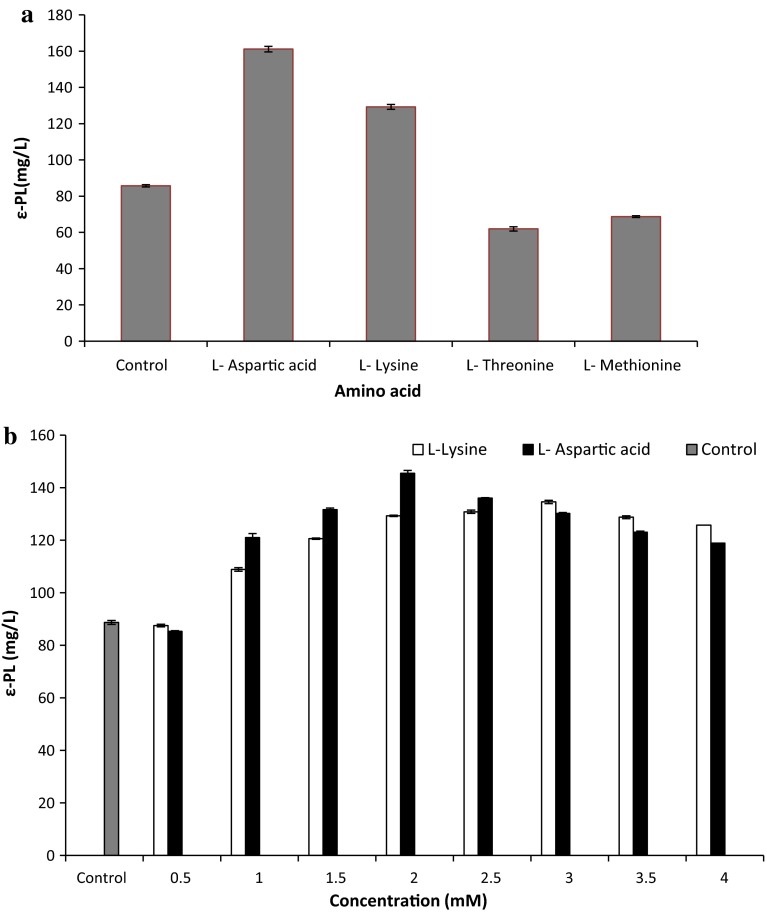
**a** Effect of amino acids on ε-PL production by *Bacillus cereus*. **b** Effect of varying concentration of l-lysine and l-aspartic acid on ε-PL production by *Bacillus cereus*

Lysine is the direct monomer precursor in the biosynthesis of ε-PL. In most bacteria, lysine is generally synthesized through aspartate pathway. Hence in the present study, the effect of Asp family amino acids on ε-PL production was studied. Figure 1a illustrates the effect of supplementation of each amino acids, viz. l-lysine, l-aspartic acid, l-methionine and l-threonine on ε-PL production by *Bacillus cereus*. It was observed that in comparison with control, addition of l-lysine and l-aspartic acid improved ε-PL yield. Highest ε-PL production was achieved in medium supplemented with l-aspartic acid (161 mg/L) followed by l-lysine (129.3 mg/L). The possible reasons for the observed results could be explained with respect to the biosynthetic pathway for ε-PL production. These amino acids act as precursors and are directly utilized in ε-PL biosynthesis, thereby enhancing its yield (Hirohara et al. 2006; Shima and Sakai 1981a). However, as compared to control low ε-PL yield was obtained with addition of l-methionine and l-threonine. This might be due to feedback inhibition of aspartate kinase and dihydrodipicolinate synthase (Anastassiadis 2007) resulting in l-isoleucine synthesis, instead of l-lysine, thereby decreasing its yield.

Subsequently, the amount of l-lysine and l-aspartic acid for ε-PL production was optimized by varying their concentration from 0.5 to 4 mM. From Fig. 1b it can be seen that with increase in concentration, maximum ε-PL yield of 145.5 mg/L was obtained with 2 mM l-aspartic acid and 134.5 mg/L with 3 mM l-lysine. Further increase in concentration resulted in decrease in ε-PL titres. This may be because at higher concentration external amino acids inhibit lysine precursor synthesis enzymes such as aspartokinase, thereby decreasing ε-PL yield (Bankar and Singhal 2011). Highest yield was achieved with 2 mM l-aspartic acid hence was selected for further studies.

### Effect of precursors on ε-PL production

**Fig. 2:**
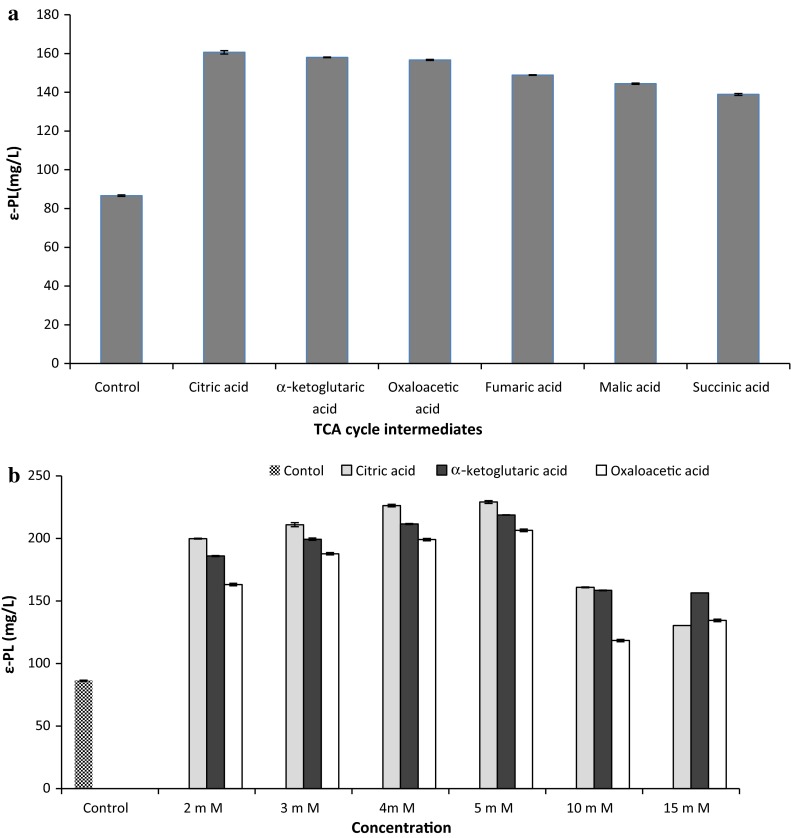
**a** Effect of precursors on ε-PL production by *Bacillus cereus*. **b** Effect of varying concentration of citric acid, α-ketoglutaric acid and oxaloacetic acid on ε-PL production by *Bacillus cereus*

TCA cycle provides precursors of certain amino acids as well as the reducing agent NADH that is used in numerous biochemical reactions. Hence effect of TCA cycle intermediates such as citric acid, α-ketoglutaric acid, oxaloacetic acid, fumaric acid, malic acid and succinic acid on ε-PL production was studied. Improved ε-PL production was observed using all TCA cycle intermediates but better yield was achieved with citric acid (160.5 mg/L), α-ketoglutaric acid (158 mg/L) and oxaloacetic acid (156 mg/L) (Fig. 2a). This might be probably due to lysine which is a direct monomer precursor in the biosynthesis of ε-PL. It is synthesized through the diaminopimelate (DAP) pathway via aspartate, produced by combining oxaloacetic acid in TCA cycle with the ammonium ion of a nitrogen source catalyzed by citric acid (Shima et al. 1983; Hamano et al. 2007; Bankar and Singhal 2011). Thus addition of metabolic precursors results in increased biosynthesis of lysine, thereby enhancing ε-PL yield. The other TCA cycle intermediates tested that is fumaric acid, malic acid and succinic acid also influenced biosynthesis of ε-PL but to a lesser extent.

Further the effect of varying concentration (2–15 mM) of citric acid, α-ketoglutaric acid and oxaloacetic acid was studied (Fig. 2b). With all the three TCA cycle intermediates used, improved yield of ε-PL was observed at 5 mM concentration. Among the three intermediates, maximum yield of 230 mg/L was obtained with 5 mM citric acid. This may be attributed to the fact that addition of citric acid facilitates the conversion of oxaloacetic acid to aspartic acid, thereby promoting ε-PL biosynthesis via Asp pathway. Above 5 mM concentration, a decrease in ε-PL production was obtained, thus explaining the concentration-dependent stimulation of ε-PL. Hence 5 mM citric acid was selected for further studies.

### Optimization of time of addition of l-aspartic acid and citric acid on ε-PL production

**Fig. 3:**
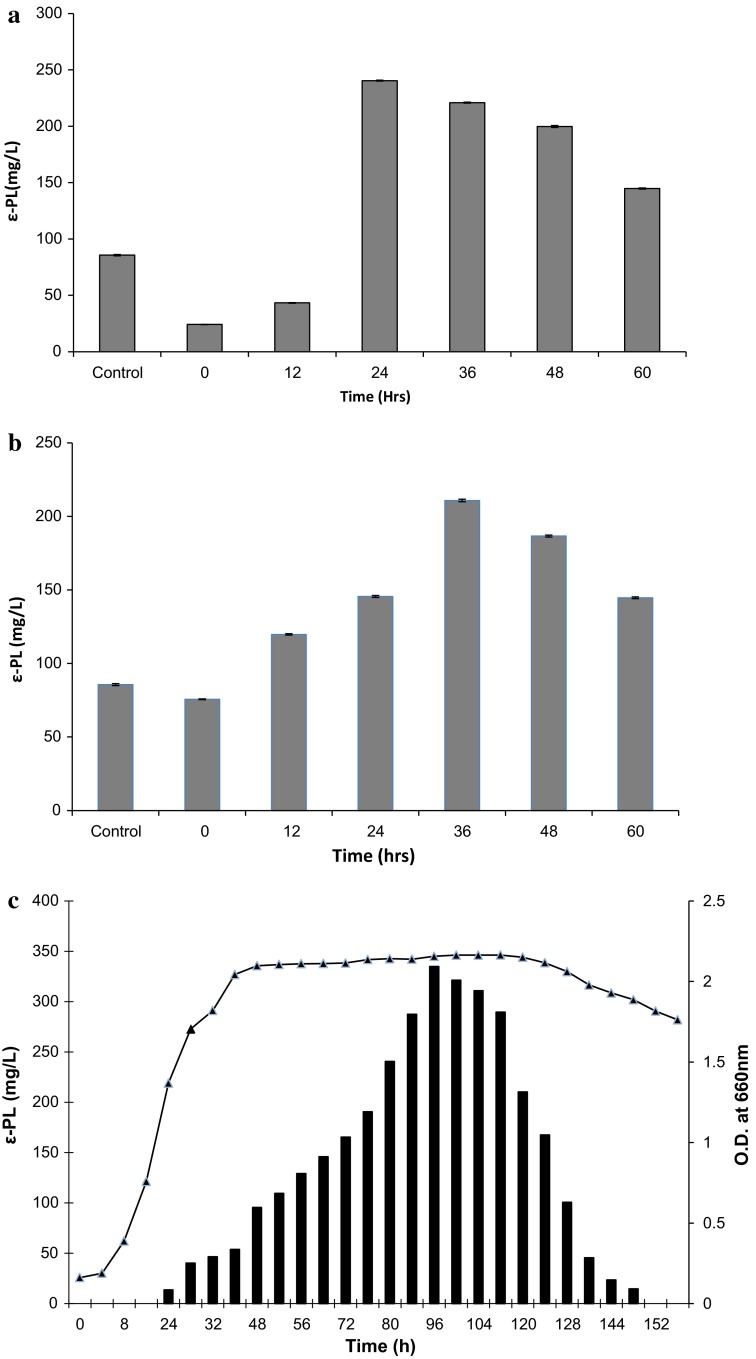
**a** Optimization of time of addition of citric acid on ε-PL production by *Bacillus cereus*. **b** Optimization of time of addition of l-aspartic acid on ε-PL production by *Bacillus cereus*. **c** Cumulative effect of l-aspartic acid and citric acid on growth profile of *Bacillus cereus* and ε-PL production (citric acid was added after 24 h and l-aspartic acid added after 36 h, respectively, in the fermentation medium)

Effect of time of addition of citric acid (Fig. 3a) and l-aspartic acid (Fig. 3b) on ε-PL was studied separately at different time intervals 12, 24, 36, 48 and 60 h from the start of fermentation process. Not much significant increase in ε-PL yield was observed with incorporation of citric acid between 0 and 12 h and aspartic acid between 0 and 24 h. This might be due to utilization of these precursors as nutritional source or due to prolonged lag phase. Thus maximum yield was attained with incorporation of citric acid in the production medium after 24 h and l-aspartic acid after 36 h from the start of fermentation process.

The next step was to study the synergistic effect of citric acid and l-aspartic acid on ε-PL production. Citric acid after 24 h and l-aspartic acid after 36 h, were incorporated in the production medium and growth profile of *Bacillus cereus* was studied (Fig. 3c). It was observed that in the lag phase from 0 to 8 h where cell growth was limited, no ε-PL was obtained. Production started after 24 h and reached maximum of 335 mg/L in 96 h corresponding to the stationary phase. After 96 h decrease in ε-PL yield was observed. Thus in comparison with control (85 mg/L), fourfold increase in ε-PL production was achieved using a combination of citric acid and l-aspartic acid in the production medium.

### Effect of intermittent feeding of glucose for ε-PL production

**Fig. 4:**
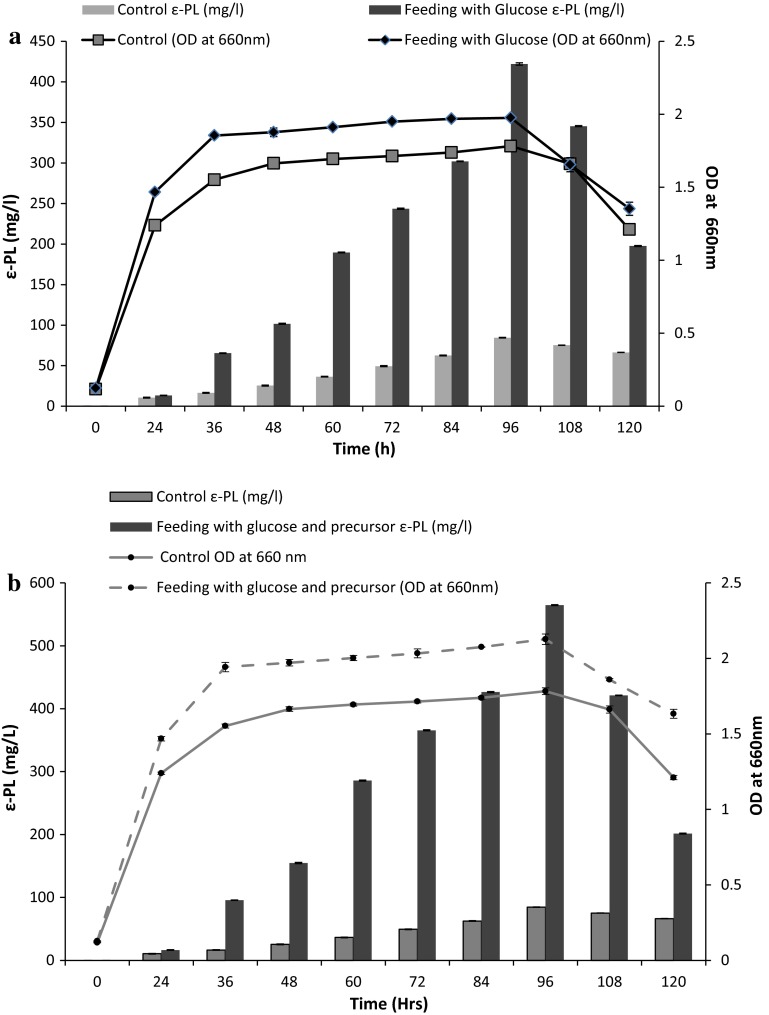
**a** Effect of intermittent feeding of glucose on ε-PL production by *Bacillus cereus.* **b** Effect of intermittent feeding of glucose in combination with metabolic precursors on ε-PL production by *Bacillus cereus*

ε-PL biosynthetic pathway most likely involves the TCA cycle that requires carbon source such as glucose for both cell growth and ε-PL production. To avoid substrate limitation and to attempt fedbatch fermentation at shake flask level, glucose was added intermittently in the production medium and its effect on ε-PL production by *Bacillus cereus* was studied (Fig. 4a). It was observed that in the control flask where no additional glucose was added in the production medium, ε-PL production started after 24 h and reached a maximum of 85 mg/L in 96 h. While in fed batch culture system, 1 mL of glucose (10 g/L) was added every 12 h and a significant increase in ε-PL concentration of 422 mg/L in 96 h was observed, thus a fivefold increase in ε-PL yield was achieved.

Initial results showed a considerable increase in ε-PL with the incorporation of citric acid and aspartic acid in the production medium. It was thought that addition of metabolic precursors in combination with intermittent glucose feeding might enhance ε-PL yield hence a combinatorial approach was attempted. In flasks where feeding strategy was employed, 1 mL (10 g/L) glucose was incorporated every 12 h in the production medium in which 5 mM citric acid was added after 24 h and 2 mM l-aspartic acid after 36 h. ε-PL production started after 24 h and a prominent increase in concentration of 565 mg/L was achieved as compared to 85 mg/L in control. More than sixfold increase in ε-PL yield was attained using *Bacillus cereus*. Thus in the present study enhanced production of ε-PL was achieved using a novel producer *Bacillus cereus* and to the best of our knowledge there are no published reports on ε-PL production using *Bacillus cereus*.

## Conclusion

In this study, ε-PL concentration was enhanced using a novel producer *Bacillus cereus*. Supplementation of the fermentation medium with 5 mM citric acid (240 mg/L) and 2 mM l-aspartic acid (210 mg/L) resulted in increased ε-PL yield as compared to control (85 mg/L). A combination of 5 mM citric acid after 24 h and 2 mM l-aspartic acid after 36 h showed a synergistic effect and further improved ε-PL concentration to 335 mg/L. An appropriate feeding strategy of intermittent glucose addition along with citric acid after 24 h and l-aspartic acid after 36 h further enhanced ε-PL to 565 mg/L. Hence, a very significant increase in ε-PL yield was achieved. Thus the present study suggests *Bacillus cereus* to be a potential alternative as an ε-PL producer.

## References

[CR1] Anastassiadis S (2007). l-Lysine fermentation. Recent Pat Biotechnol.

[CR2] Bankar SB, Singhal RS (2011). Metabolic precursors enhance the production of poly(ε-l-lysine) by *Streptomyces noursei* NRRL 5126. Eng Life Sci.

[CR3] Bankar SB, Singhal RS (2013). Panorama of poly-ε-lysine RSC Adv.

[CR4] Banker SB, Singhal RS (2010). Optimization of poly-ε-lysine production by *Streptomyces noursei* NRRL 5126. Bioresour Technol.

[CR5] Chedda AH, Vernekar MR (2014). Improved production of natural food preservative ε-poly-l-lysine using a novel producer *Bacillus cereus*. Food Bioscience.

[CR6] El-Sersy N, Abdelwahab A, Abouelkhiir S, Abouzeid D, Sabry S (2012). Antibacterial and anticancer activity of ε-poly-l-lysine (ε-PL) produced by a marine *Bacillus subtilis* sp.. J Basic Microbiol.

[CR7] Hamano Y, Nicchu I, Shimizu T, Onji Y, Hiraki J, Takagi H (2007). ε-Poly-l-Lysine producer *Streptomyces albulus*, has feedback inhibition resistant aspartokinase. Appl Biochem Biotechnol.

[CR8] Hirohara H, Takehara M, Saimura M, Masayuki A, Miyamoto M (2006). Biosynthesis of poly(ε-l-lysine)s in two newly isolated strains of *Streptomyces* sp.. Appl Microbiol Biotechnol.

[CR9] Kahar P, Iwata T, Hiraki J, Park EY, Okabe M (2001). Enhancement of ε-polylysine production by *Streptomyces albulus* strain 410 using pH control. J Biosci Bioeng.

[CR10] Kobayashi K, Nishikawa M (2007). Promotion of ε-poly-l-lysine production by iron in *Kitasatospora kifunense*. World J Microbiol Biotechnol.

[CR11] Li S, Tang L, Chen X, Liao L, Li F, Mao Z (2010). Isolation and characterization of a novel ε-poly-l-lysine producing strain: *Streptomyces griseofuscus*. J Ind Microbiol Biotechnol.

[CR12] Ouyang J, Xu H, Li S, Zhu H, Chen W, Zhou J, Wu Q, Xu L, Ouyang P (2006). Production of ε-poly-l-lysine by newly isolated *Kitasatospora* sp. PL6-3. Biotechnol J.

[CR13] Saimura M, Takehara M, Mizukami S, Kataoka K, Hirohara H (2008). Biosynthesis of nearly monodispersed poly(ε-l-lysine) in *Streptomyces* sp.. Biotechnol Lett.

[CR14] Shen WC, Yang D, Ryser HJ (1984). Colorimetric determination of microgram quantities of polylysine by trypan blue precipitation. Anal Biochem.

[CR15] Shih IL, Van YT, Shen MH (2004). Biomedical applications of chemically and microbiologically synthesized poly(glutamic acid) and poly(lysine). Mini Rev Med Chem.

[CR16] Shih IL, Shen MH, Van YT (2006). Microbial synthesis of poly (ε-lysine) and its various applications. Bioresour Technol.

[CR17] Shima S, Sakai H (1977). Polylysine produced by *Streptomyces*. Agric Biol Chem.

[CR18] Shima S, Sakai H (1981). Poly-l-lysine produced by *Streptomyces.* Part II. Taxonomy and fermentation studies. Agric Biol Chem.

[CR19] Shima S, Sakai H (1981). Poly-l-lysine produced by *Streptomyces.* Part III. Chemical studies. Agric. Biol. Chem.

[CR20] Shima S, Oshima S, Sakai H (1983). Biosynthesis of ε-poly-l-lysine by washed mycelium of *Streptomyces albulus* no-346. Nippon Nogeikagaku Kaishi.

[CR21] Shima S, Matsuoka H, Iwamoto T, Sakai H (1984). Antimicrobial action of ε-poly-l-lysine. J Antibiot.

[CR22] Shukla SC, Mishra A (2013). ε-Polylysine production from sugar cane molasses by a new isolates of *Bacillus* sp. and optimization of the fermentation condition. Ann Microbiol.

[CR23] Takehara M, Hibino A, Saimura M, Hirohara H (2010). High yield production of short chain length poly (ε-l-lysine) consisting of 5–20 residues by *Streptomyces aureofaciens* and its antimicrobial activity. Biotechnol Lett.

[CR24] Wang G, Jia S, Wang T, Chen L, Song Q, Li W (2011). Effect of Ferrous ion on ε-poly-l-lysine biosynthesis by *Streptomyces diastatochromogenes* CGMCC3145. Curr Microbiol.

[CR25] Zhang Y, Feng XH, Xu H, Yao Z, Ouyang PK (2010). ε-Poly-l-Lysine production by immobilized cells of *Kitasatospora* sp. MY 5–36 in repeated fed-batch cultures. Bioresour Technol.

